# Clinical Utility of Fluid Volume Assessment in Heart Failure Patients Using Bioimpedance Spectroscopy

**DOI:** 10.3389/fcvm.2021.636718

**Published:** 2021-04-07

**Authors:** Andrew J. Accardi, Bradley S. Matsubara, Richelle L. Gaw, Anne Daleiden-Burns, James Thomas Heywood

**Affiliations:** ^1^Department of Emergency Medicine, Scripps Memorial Hospital Encinitas, Encinitas, CA, United States; ^2^ImpediMed, Inc., Carlsbad, CA, United States; ^3^ImpediMed Limited, Brisbane, QLD, Australia; ^4^Heart Failure Recovery and Research Program, Scripps Memorial Hospital La Jolla, La Jolla, CA, United States

**Keywords:** heart failure, bioimpedance spectroscopy, extracellular fluid, total body water, case study

## Abstract

**Background:** Bioimpedance spectroscopy (BIS) is a non-invasive method used to measure fluid volumes. In this report, we compare BIS measurements from patients with heart failure (HF) to those from healthy adults, and describe how these point-of-care fluid volume assessments may be applied to HF management.

**Methods and results:** Fluid volumes were measured in 64 patients with NYHA class II or III HF and 69 healthy control subjects. BIS parameters including extracellular fluid (ECF), intracellular fluid (ICF), total body water (TBW), and ECF as a percentage of TBW (ECF%TBW) were analyzed. ECF%TBW values for the HF and control populations differed significantly (49.2 ± 3.2% vs. 45.2 ± 2.1%, respectively; *p* < 0.001); both distributions satisfied criteria for normality. Interquartile ranges did not overlap (46.7–51.0% vs. 43.8–46.4%, respectively; *p* < 0.001). Subgroup analyses of HF patients who underwent transthoracic echocardiography showed that impedance measurements correlated with inferior vena cava size (Pearson correlation −0.73, *p* < 0.0001). A case study is presented for illustrative purposes.

**Conclusions:** BIS-measured ECF%TBW values were significantly higher in HF patients as compared to adults without HF. We describe three strata of ECF%TBW (normal, elevated, fluid overload) that may aid in clinical risk stratification and fluid volume monitoring of HF patients.

**Clinical Trial Registration:** COMPARE – www.ClinicalTrials.gov; IMPEL – www.ClinicalTrials.gov; Heart Failure at Home – www.ClinicalTrials.gov, identifier: NCT02939053; NCT02857231; NCT04013373.

## Introduction

Heart failure (HF) affects ~26 million people worldwide, with the prevalence increasing as the population ages (1). In the United States alone, HF affects an estimated 6.2 million individuals (2). This condition places a substantial burden on health care systems with high rates of hospitalizations, readmissions, and outpatient visits. Despite advances in treatment and monitoring, HF-related mortality remains high (1). Patients with stable ventricular function and unchanged medications can still decompensate, resulting in recurrent hospitalizations (3). Once hospitalized, up to 25% of HF patients are readmitted within 30 days (4, 5).

Bioimpedance spectroscopy is a non-invasive method used to assess fluid volume status. The electrical impedance of biological tissue is measured in response to an alternating current across a spectrum of 256 frequencies. An electrical current applied to the body will conduct primarily through fluid due to its low resistivity (6). Impedance values are then used to quantify intracellular fluid (ICF), extracellular fluid (ECF), and total body water (TBW), as well as other fluid and tissue parameters (7). BIS has enabled improved discrimination of fluid overload from HF as a cause of dyspnea, and is sensitive to changes in both pulmonary and peripheral edema (8–12). In addition, BIS measurements of ECF (13, 14) and TBW (14–18) have also been shown to correlate strongly with gold-standard bromide and deuterium oxide dilution methods, respectively (Table 1). BIS measurements have also been shown to correlate well with echocardiographic indicators of fluid overload (inferior vena cava size, right atrial pressure, and pulmonary artery systolic pressure) (19). The purpose of this report is to compare point-of-care bioimpedance spectroscopy (BIS) measurements from patients with HF to those from healthy adults, and to describe the range of BIS-derived ECF%TBW values in a clinically relevant way.

**Table 1:** ECF and TBW correlation coefficients for BIS measurements vs. gold-standard dilution techniques.

**First author, publication year [reference]**	**Study population**	**Correlation coefficient**
**Correlation between extracellular fluid (ECF) measured by bioimpedance spectroscopy (BIS) and bromide dilution**		
Birzniece, 2015 (13)	Healthy athletes	*r* = 0.84
Van De Ham, 1999 (14)	Renal transplant patients	*r* = 0.87
**Correlation between total body water (TBW) measured by bioimpedance spectroscopy (BIS) and deuterium oxide dilution**		
Cicone, 2019 (15)	Healthy individuals	*r* = 0.93
Kerr, 2015 (16)	Resistance trained individuals	*r* = 0.90
Moon, 2009 (17)	Overfat and obese individuals	*r* = 0.96
Moon, 2008 (18)	Healthy individuals	*r* = 0.98
Van De Ham, 1999 (14)	Renal transplant patients	*r* = 0.94

## Materials and Methods

### Clinical Study Participants

In order to characterize BIS parameters in individuals with and without HF, observational data from six clinical studies utilizing BIS were evaluated (years of data collection: 2017-2019). A total of 64 patients with New York Heart Association (NHYA) Class II or III HF were enrolled across three clinical studies and combined to form a population for HF patients (HF-pop): two patients (ClinicalTrials.gov identifier NCT02939053) were clinically stable NYHA Class III men with CardioMEMS pulmonary artery pressure monitors who performed daily BIS measurements at home for 30 days; 12 patients (NCT02857231) were clinically stable and had BIS measurements taken two or three times per week in an outpatient advanced HF clinic over a 30-day period; 50 patients (NCT04013373) were enrolled within 72-h after discharge from a hospitalization for acute decompensated HF and took daily BIS measurements at home over 45 days. A total of 69 self-reported healthy control subjects aged 40 years or more were enrolled across three different clinical studies and combined to form a control population (CON-pop). A summary of these populations is provided in Table 2.

**Table 2:** Clinical studies enrolling HF patients and healthy control subjects.

**Clinical study description**	***N***	**Gender (female, male)**	**Age (years)**
New York Heart Association Class III HF patients measured daily at home over 30 days	2	0F, 2M	70.5 ± 2.1
New York Heart Association Class III HF patients measured 3 times per week in clinic over 30 days	12	5F, 7M	65.0 ± 15.6
New York Heart Association Class II and III HF patients recently discharged from hospitalization due to decompensated HF measured daily at home for 45 days	50	23F, 27M	70.2 ± 15.1
Healthy university population 40 years or older measured at a single clinic visit	13	8F, 5M	48.8 ± 8.8
Healthy university population 40 years or older measured at a single clinic visit	25	11F, 14M	47.9 ± 9.7
Healthy general population 40 years or older measured at a single clinic visit	31	18F, 13M	57.8 ± 11.3
**Combined populations**	***N***	**Gender (female, male)**	**Age (years)**
Heart Failure Patients (HF-pop)	64	28F, 36M	69.3 ± 14.8
Healthy Control Subjects (CON-pop)	69	37F, 32M	52.5 ± 11.2

All contributing clinical studies received the approval of an Independent Review Board (IRB) or Ethics Committee (EC), and all participants provided written informed consent. Per BIS device instructions for use, individuals were excluded if they were amputees, had metallic implants, or implanted devices such as pacemakers or implantable cardioverter defibrillators (ICDs). Potential subjects were also excluded if they were pregnant, breast feeding, or had other comorbidities that could result in fluid overload; namely, renal failure (dialysis dependent at the time of enrollment), nephrotic syndrome or nephrosis, lymphedema, chronic liver failure or cirrhosis, and thrombophlebitis or deep vein thrombosis of the extremities (within 90 days prior to enrollment).

### Bioimpedance Spectroscopy Measurements

**Figure 1:**
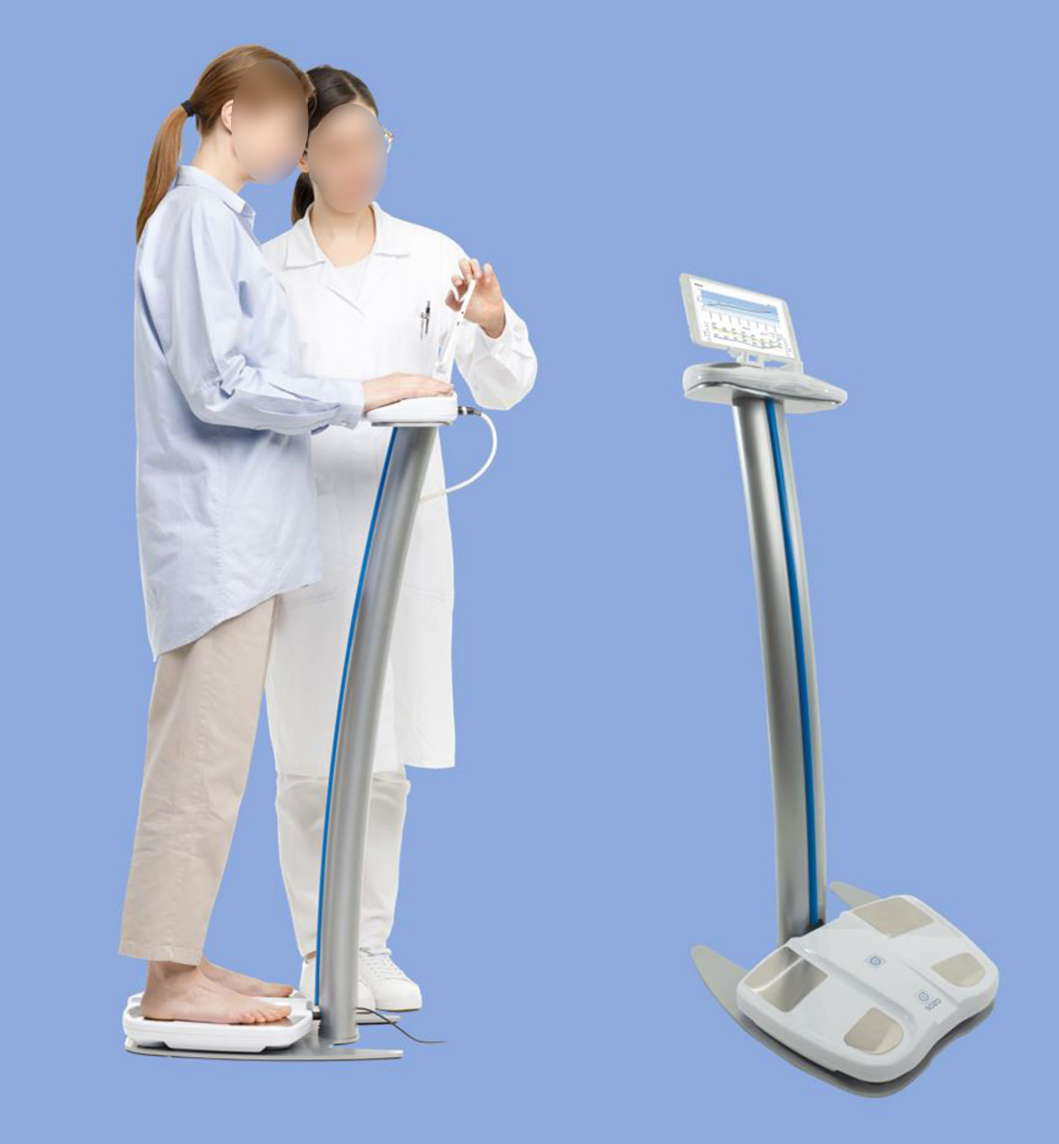
SOZO device. As shown, the device is configured to perform bioimpedance spectroscopy (BIS) measurements with the subject in a standing position. Bare hands and feet must be in direct contact with the electrodes (i.e., no shoes, socks, stockings, or gloves), and metallic/electronic items should be removed. BIS measurement and fluid status reporting takes ~30 s.

BIS measurements were performed using the SOZO device (ImpediMed Limited, Brisbane, Australia). The device (Figure 1) measures the resistance and reactance at 256 frequencies from 3 to 1,000 kilohertz (kHz). It is a mains-powered device that takes octopolar measurements using stainless-steel hand and foot plates in a standing or seated position. A measurement takes ~30 s and is performed at the point-of-care. BIS has been used to assess small changes in lymphatic fluid in order to detect subclinical lymphedema in cancer survivors (20–23). Other applications include use in venous insufficiency, kidney failure, and evaluation of malnutrition/hydration status (24–27). The SOZO system has been cleared by the United States Food and Drug Administration (FDA) for use in monitoring fluid in HF patients.

In each study, BIS measurements were simultaneously taken of both arms, both legs, and right and left sides of the body as per the manufacturer's instructions for use. All participants were weighed using digital scales to the nearest 0.1 kg, and had their height recorded to the nearest centimeter using a wall or stand-mounted stadiometer. In the case of the HF-pop patients, multiple measurements were taken either daily at home or several times per week in a clinic over a monitoring period of up to 45 days. All CON-pop measures were taken in triplicate during a single clinic visit.

### Transthoracic Echocardiography

A subgroup of 12 HF-pop patients enrolled in the IMPEL clinical study (ClinicalTrials.gov identifier NCT02857231) underwent transthoracic echocardiography (TTE). At each clinic visit, limited TTE was performed by licensed echocardiographers and reviewed by Board-certified cardiologists to obtain echocardiographic measurements of inferior vena cava (IVC) size and estimates of right atrial pressure (RAP). According to recommendations by the American Society of Echocardiography (ASE), RAP values were categorized into three groups: group 1 included any RAP below 8 mmHg, group 2 included values of 8-14.99 mmHg, and group 3 included all values equal to 15 mmHg. The ASE has defined a normal RAP as 3 mmHg, intermediate as 8 mmHg, and high as 15 mmHg (19).

### Data Processing and Statistical Analysis

Prior to analysis, all BIS measurements were reviewed for suitable quality. This was done by assessing the quality of the fit of the raw impedance data to the recognized semi-circular Cole plot of biological tissue (28). Only data that met pre-defined criteria for measurement quality were used to calculate R_0_ (the resistance of ECF, at theoretical 0 kHz) and R_inf_ (the resistance of TBW, at theoretical infinite kHz) for each measure. These values were converted to absolute ECF and TBW volumes using the Hanai mixture theory implemented in the manufacturer's software and then the ECF%TBW was calculated. ECF and TBW are calculated independently (using R_0_ and R_inf_, respectively); as such, the use of ECF/TBW expressed as a percentage allows for indexing.

Because the number of and interval between BIS measurements taken during clinical studies varied, the data was standardized to include one representative measurement per subject. Based on previous work which demonstrated low variability in a healthy population over time, the average of 3 measures in a single clinic visit was used for the healthy control individuals (22). The HF patients were tracked over multiple days with multiple measurements; to mitigate issues associated with repeated measures per patient, the median BIS value for each HF patient was used.

Calculations were performed using MedCalc version 11.6.1.0. Unless otherwise specified, results are presented as means ± standard deviations, and/or medians with quartiles and ranges. Statistical significance was defined as a *p*-value < 0.05.

Plots of ECF%TBW, ECF, ICF, TBW, and patient weight vs. time are presented for a patient enrolled in the Heart Failure at Home study (ClinicalTrials.gov NCT04013373). The timeline is annotated for symptoms, signs, medication changes, and significant clinical events (e.g., rehospitalizations for HF) and is presented in Figure 5. Investigators were blinded to BIS values, so management was per standard of care.

## Results

Participant age, physical characteristics, and BIS-derived fluid volumes are summarized in Table 3. HF-pop patients were significantly older than CON-pop subjects (median ages 71.4 and 50.0 years, respectively; *p* < 0.001), and had significantly higher body mass indices (BMI, 29.5 ± 6.1 vs. 25.9 ± 4.0 kg/m^2^, respectively; *p* = 0.0001). There were no significant differences in ICF or TBW.

**Table 3:** Age, physical characteristics, and bioimpedance spectroscopy measurements.

	**Healthy control subjects**						**Heart failure patients**						***P*-value**
	***N*** **=** **69 (32 males, 37 females)**						***N*** **=** **64 (36 males, 28 females)**						
		**Quartiles**						**Quartiles**					
	**Mean ± SD**	**Min**	**25th**	**Median**	**75th**	**Max**	**Mean ± SD**	**Min**	**25th**	**Median**	**75th**	**Max**	
Age (years)	52.5 ± 11.2	40.0	43.0	50.0	61.0	77.0	69.3 ± 15.0	28.0	59.9	71.4	79.6	96.0	<0.001
Height (cm)	171.6 ± 8.2	150.5	165.1	171.5	177.8	190.5	167.8 ± 11.7	147.3	157.5	167.6	177.8	188.0	0.0347
Weight (kg)	76.6 ± 15.0	46.0	65.9	75.4	84.5	121.3	83.2 ± 19.0	37.6	70.8	80.3	99.2	133.8	0.0263
BMI (kg/m^2^)	25.9 ± 4.0	17.9	23.3	25.6	27.6	39.4	29.5 ± 6.1	17.3	25.0	28.7	33.3	49.5	0.0001
Body R_0_ (Ohms)	664.8 ± 97.6	486.0	602.7	648.6	735.6	927.6	591.4 ± 121.4	363.0	503.7	561.1	663.6	928.7	0.0002
Body R_inf_ (Ohms)	491.9 ± 81.4	347.4	426.0	488.8	540.8	715.0	470.4 ± 103.9	306.0	394.0	453.5	525.3	835.6	0.1881
ECF (liters)	18.2 ± 4.1	11.7	14.9	18.0	20.7	29.5	20.0 ± 5.4	8.9	15.3	19.5	24.1	33.2	0.0299
ICF (liters)	22.0 ± 4.5	14.0	18.8	21.1	25.7	36.3	20.6 ± 5.2	7.8	16.4	19.9	23.8	33.6	0.0912
TBW (liters)	40.2 ± 8.4	25.8	33.7	38.0	46.2	65.7	40.6 ± 10.2	16.6	32.6	39.9	48.2	63.8	0.8046
ECF%TBW (%)	45.2 ± 2.1	41.5	43.8	44.8	46.4	50.0	49.2 ± 3.2	43.2	46.7	48.8	51.0	56.5	<0.001
ICF%TBW (%)	54.8 ± 2.1	50.0	53.6	55.2	56.2	58.5	50.8 ± 3.3	43.5	48.9	51.2	53.3	56.9	<0.001

*BMI, body mass index; R_0_, resistance at zero Hertz; R_inf_, resistance at infinite Hertz; ECF, extracellular fluid; ICF, intracellular fluid; TBW, total body water; ECF%TBW, extracellular fluid as a percentage of total body water; ICF%TBW, intracellular fluid as a percentage of total body water*.

Table 3 shows that significant differences (*p* < 0.05) exist between the CON-pop and HF-pop for body R_0_, ECF, ECF%TBW, and ICF as a percentage of TBW (ICF%TBW) measures. Given that clinicians are familiar with the ECF%TBW metric and the fact there is published use of this parameter, it was further analyzed. Baseline systemic blood pressure, heart rate, and concomitant medications are summarized in Table 4.

**Table 4:** Baseline systemic blood pressure, heart rate, and concomitant medications for heart failure patients.

Parameter (*n* = 61)^*^	**Mean ± SD**
Systolic blood pressure (mmHg)	120.0 ± 16.8
Diastolic blood pressure (mmHg)	71.1 ± 12.4
Heart rate (beats per minute)	76.9 ± 12.3
**Concomitant medication (*****n*** **=** **63)^∧^**	**Count (percentage)**
ACEI/ARB	24 (38%)
Digoxin	8 (13%)
Beta-blocker	53 (84%)
HCN channel blocker	3 (5%)
Sacubitril/Valsartan	7 (11%)
MRA	27 (43%)
Diuretic	52 (83%)

* *Not available for three patients*.

∧ *Not available for one patient*.

*mmHg, millimeters of mercury; SD, standard deviation; ACEI, angiotensin converting enzyme inhibitor; ARB, angiotensin receptor blocker; HCN, hyperpolarization-activated cyclic nucleotide-gated; MRA, mineralocorticoid receptor antagonist*.

### Extracellular Fluid as a Percentage of Total Body Water

**Figure 2:**
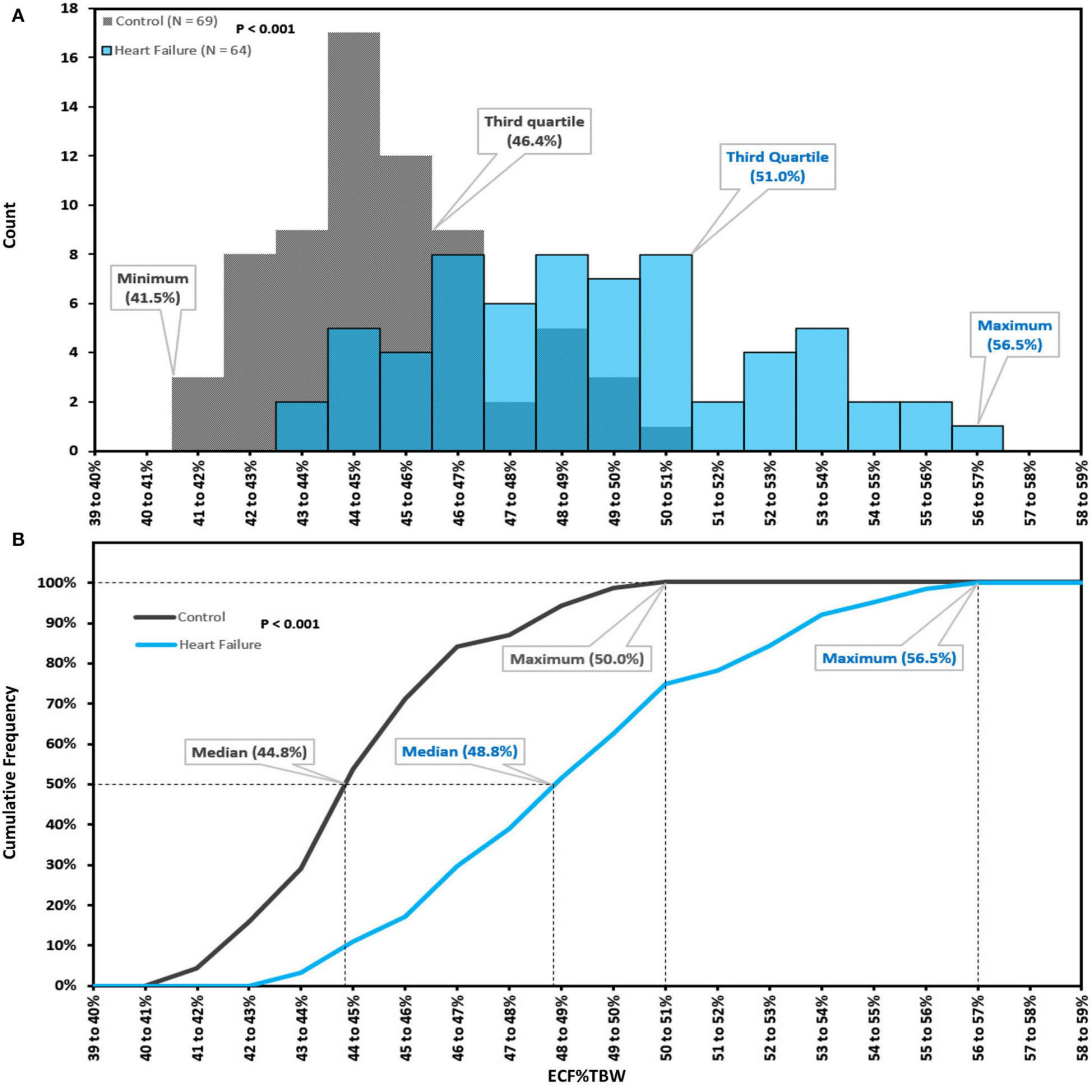
Extracellular fluid percentage of total body water; histogram **(A)**, cumulative frequency curves **(B)**. Extracellular fluid percentage of total body water (ECF%TBW) for Healthy Control Subjects (CON-pop, *N* = 69, shown in gray) and Heart Failure Patients (HF-pop, *N* = 64, shown in blue); histogram **(A)**, and cumulative frequency curves **(B)**.

**Figure 3:**
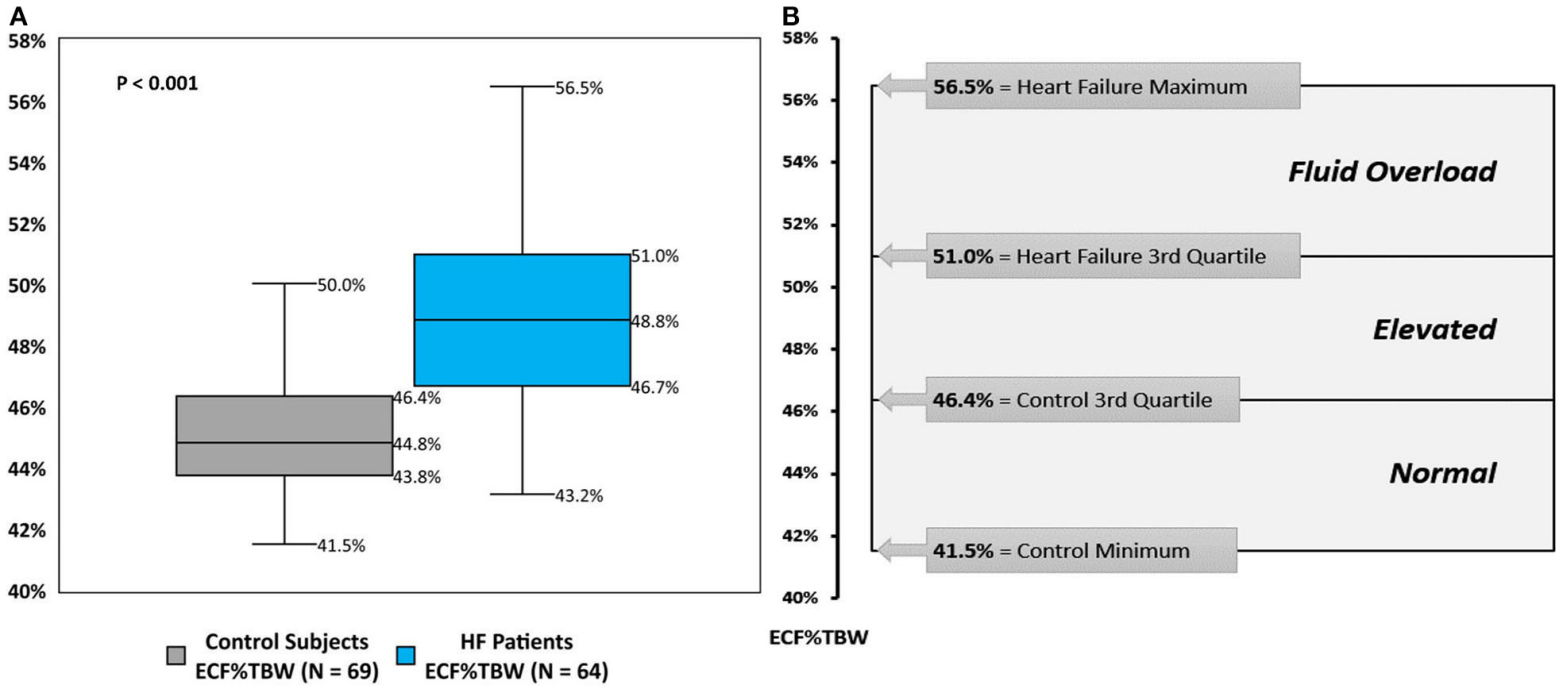
Extracellular fluid percentage of total body water; box-and-whisker plots **(A)**, and clinical strata **(B)**. **(A)** box-and-whisker plots for bioimpedance spectroscopy-derived extracellular fluid percentage of total body water (ECF%TBW) for Healthy Control Subjects (CON-pop, gray plot) and Heart Failure Patients (HF-pop, blue plot). **(B)** Normal, bottom three CON-pop quartiles; Elevated, bound by CON-pop 3rd quartile and HF-pop 3rd quartile; Fluid Overload, highest HF-pop quartile.

The distribution of BIS-derived ECF%TBW measurements for both CON-pop and HF-pop satisfied criteria for normality (Chi-square test, *P* = 0.4623 and *P* = 0.9262, respectively) (29). ECF%TBW was significantly higher for HF-pop as compared to CON-pop (49.2 ± 3.2% vs. 45.2 ± 2.1%, respectively; *p* < 0.001); interquartile ranges did not overlap (46.7–51.0% vs. 43.8–46.4%, respectively; *p* < 0.001). These distributions are shown graphically in Figure 2 (histogram and cumulative frequency curves) and Figure 3A (box-and-whisker plots). Based on these distributions, three clinical strata of ECF%TBW are shown in Figure 3B. The bottom three CON-pop quartiles define the “Normal” stratum (41.5–46.4%), “Fluid Overload” is defined by the highest HF-pop quartile (51.0–56.5%), and the “Elevated” stratum falls in between. Of note, the CON-pop maximum was 50.0%, so no healthy subject's ECF%TBW measurement exceeded the 51.0% threshold for fluid overload.

### Echocardiographic Subgroup Analysis

**Figure 4:**
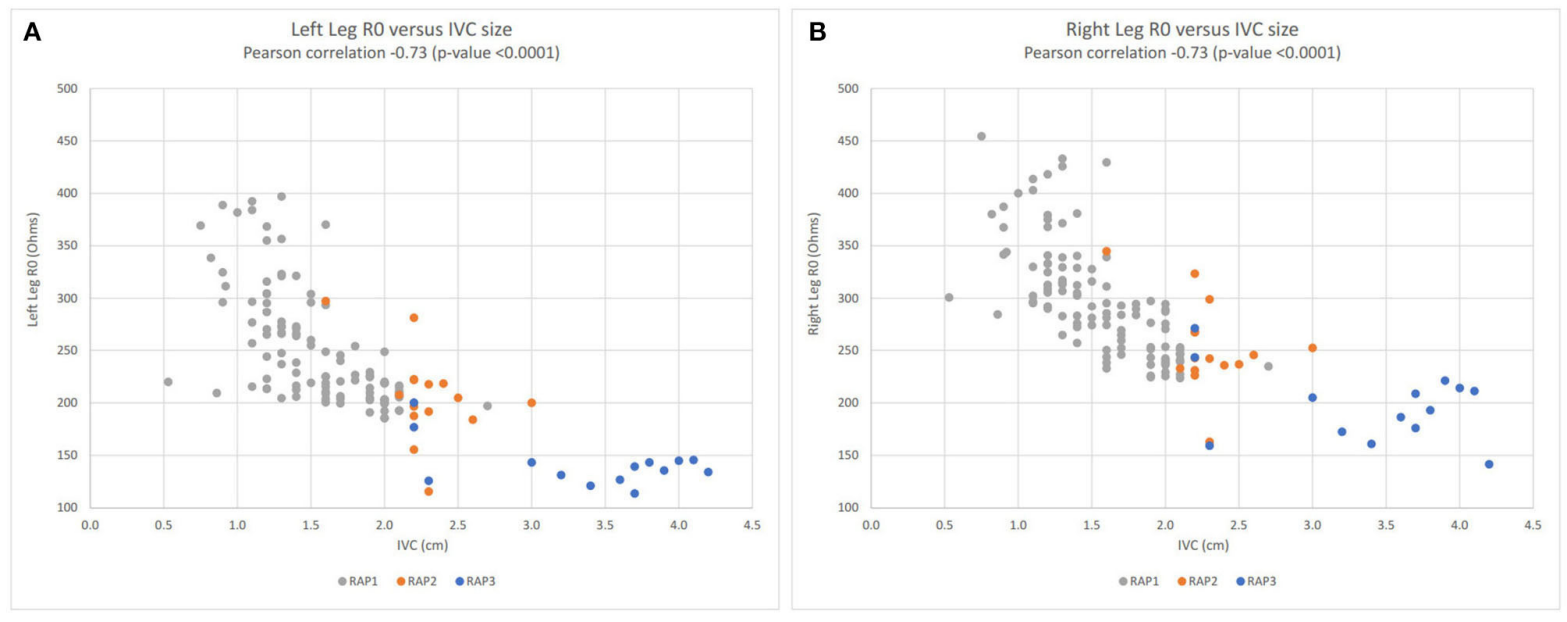
Lower extremity R_0_ impedance measurements vs. inferior vena cava (IVC) size; left leg **(A)**, right leg **(B)**. Left leg **(A)** and right leg **(B)** scatter plots for R_0_ impedance vs. inferior vena cava (IVC) size for the subgroup of 12 heart failure patients enrolled in the IMPEL clinical study (ClinicalTrials.gov identifier NCT02857231). Right atrial pressure (RAP) categories: RAP1 in gray (<8 mmHg), RAP2 in orange (8–14.99 mmHg), and in blue RAP3 (15 mmHg). R_0_ impedance is inversely related to extracellular fluid volume. Hence, in both legs, lower impedance values are associated with larger IVC size and higher right atrial pressures. R_0_, resistance at zero Hertz; IVC, inferior vena cava; RAP, right atrial pressure; cm, centimeter.

The subgroup analysis of IMPEL clinical study patients (ClinicalTrials.gov identifier NCT02857231) is presented in Figure 4. These 12 HF patients underwent serial (two or three times weekly) transthoracic echocardiography (TTE). Both left and right leg R_0_ impedance measurements were correlated with inferior vena cava size (Pearson correlation −0.73, *p*-value < 0.0001 for each leg) and TTE categories of estimated right atrial pressure (RAP).

### Clinical Case Study (Figure 5)

This patient is an 87 year-old man with NYHA class III heart failure with reduced left-ventricular ejection fraction (35–40%), and a history of hypertension, atrial fibrillation, and chronic kidney disease. ECF%TBW was markedly elevated (56.7%) upon study entry, and the patient was readmitted to the hospital on study day 5. After a skilled nursing facility (SNF) stay, home monitoring resumed on study day 30. His ECF%TBW remained very high (57.0%), and bumetanide dose increases between study days 30 and 49 had minimal effect on fluid volumes and weight. Metolazone was started on study day 64, and the patient responded with a reduction in ECF%TBW to 52.7% on study day 76. He then left the study briefly only to be re-enrolled after his second readmission for heart failure. When BIS measurements resumed on study day 87, his ECF%TBW had risen to 54.7%. Metolazone therapy was reinitiated, and clinical, fluid volume, and weight stability was finally achieved by study day 115. The ECF%TBW strata shown in Figure 3B are based on population data and should be interpreted in clinical context. This patient was almost exclusively >51% (fluid overloaded state); achieving normal fluid status (41.5–46.4%) is an unrealistic goal in this case. With unblinded, real-time BIS data, it's likely that his caregivers would have recognized persistent fluid overload at the time of study entry as his ECF%TBW was 56-57%, markedly elevated even for this patient. An ECF%TBW of 50% and an ECF volume of 16 liters turned out to be reasonable targets in this case; a future goal of fluid monitoring in HF would be to identify and maintain target fluid volumes more quickly than is currently possible with weight tracking alone.

**Figure 5:**
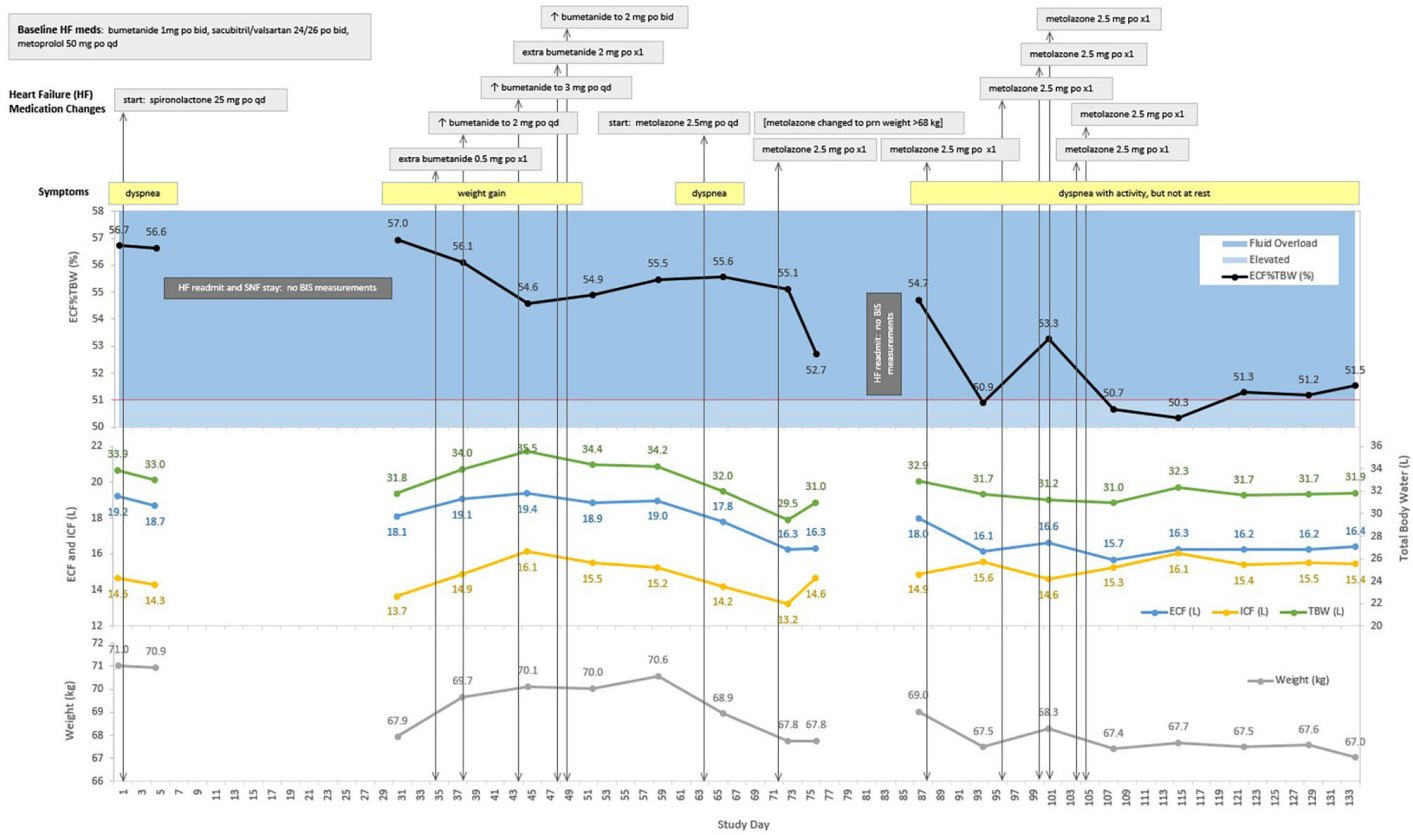
Case Study: 87 year-old man with two heart failure readmissions. ECF, extracellular fluid; ICF, intracellular fluid; TBW, total body water; ECF%TBW, extracellular fluid as a percentage of total body water; mg, milligram; po, oral; qd, daily; L, liters; kg, kilograms; BIS, bioimpedance spectroscopy; SNF, skilled nursing facility. The red line at 51% ECF%TBW indicates the transition from elevated fluid volume to fluid overload.

## Discussion

Monitoring strategies and development of novel markers to guide HF management remain elusive (30, 31). Current standards of care to assess volume status include monitoring patient weight, physical exam findings, and resolution of symptoms. These methods are often insensitive and may not provide adequate warning of impending decompensation (32). Implanted pulmonary artery pressure monitoring systems have been shown to decrease rates of hospitalization and improve quality of life (33–35), but require an invasive procedure for implantation with associated risks and cost. Accurate tracking of fluid volume fluctuations has been shown to be helpful for the individualized management of diuretic therapy, which remains a cornerstone of HF management (36, 37).

Formulae for calculating estimated plasma volume status (ePVS) have been shown to correlate well with gold-standard radioisotope assay measures of plasma volume (PV). Examples include Strauss' formula (for change in ePVS), Duarte's formula [ePVS = (100 – hematocrit (%) / hemoglobin (g/dL)], and the Hakim formula [ePVS = ((actual PVS – ideal PV) / ideal PV) ^*^ 100]. These calculations utilize hematocrit, hemoglobin, and body weight and thereby avoid the complex, costly, and logistically challenging radioisotope quantification of PV. Reliance upon “dry” body weight—which is difficult to measure in the setting of heart and/or kidney failure—is a potential limitation of the Hakim formula. Associations of ePVS with clinical outcomes in heart failure were recently reviewed by Kobayashi et al. (38) who conclude that initial data are encouraging and warrant investigation in adequately powered prospective clinical trials.

Bedside lung ultrasound (LUS) is a relatively new method used to assess pulmonary congestion. Sonographic evaluation of the antero-lateral chest can detect extravascular lung fluid imaged as “B-lines.” Mottola et al. (39) used LUS to evaluate pulmonary edema in a single-center observational study of 36 patients during the early post-operative period following kidney transplant surgery. Horton and Collins (40) suggest that LUS may help discriminate between cardiogenic and non-cardiogenic causes of dyspnea in the emergency department.

In the present report, based on measurements from HF and healthy control populations, we describe three strata of BIS-measured ECF%TBW (Figure 3B) that may contribute to clinical risk stratification and may serve as a tool to help facilitate future outcomes research. BIS is rapid (~30 s per measurement) and non-invasive, so results can be used in real-time to assist with clinical decision making at the bedside, in the clinic, and potentially at home. Real-time availability is not practical with traditional ECF%TBW determination methods such as DEXA that requires a scan with ionizing radiation, and heavy water or bromide dilution that require special reagents and blood draws. This report is not intended to directly compare BIS to these techniques, but rather to describe a clinically relevant way to quantify fluid volume status.

By way of comparison to previously published data, mean and standard deviation values of ECF%TBW from this report's control population (45.2 ± 2.1%) are in keeping with National Health and Nutrition Examination Survey (NHANES) reference data for adults aged 50-59 years (47.2 ± 2.0% for women, and 41.7 ± 1.6% for men) (41). Additionally, our 51.0% BIS-derived ECF%TBW threshold for fluid overload closely approximates the 50.0% cut-off defined by Sergi et al. who used gold-standard methods of DEXA, deuterium oxide dilution, and bromide dilution. In their publication, ECF%TBW values in excess of 50.0% were independently associated with a 10-fold higher likelihood of fluid retention (odds ratio of 10, with 95% confidence interval 3.3–30.3) (42). Indeed, the highest control population ECF%TBW value we measured was 50.0%; this provides further justification for the 51% BIS-based ECF%TBW threshold for fluid overload.

Liu et al. describe 6-month prognostic value for multi-frequency bioelectrical impedance analysis (MFBIA) in patients hospitalized for acute HF using an ECF/TBW cut-off value of 0.390 (39.0%); this so-called “edema index” was derived from 6-frequency MFBIA performed in 58 HF patients (43). We used a BIS technique that measures impedance over a spectrum of 256 frequencies thereby enabling Cole analysis for more accurate determination of R_0_ and R_inf_, and therefore more accurate ECF, TBW, and ECF%TBW (44). BIS provides a more direct, individualized measure of ECF and TBW than other bioimpedance approaches (45). This difference in measurement technique likely accounts for the discrepancy in thresholds for fluid overload between MFBIA and BIS.

We found other BIS-derived parameters, such as R_0_ and ECF, showed statistically significant differences between HF and control populations (Table 3). In our TTE subgroup analysis, R_0_ measurements from both lower extremities were shown to correlate well with TTE-measured IVC size (Pearson correlation−0.73, Figure 4) and estimated right atrial pressure, metrics that are used clinically to evaluate preload and filling pressure. This suggests that BIS may be able to provide similar information without the cost, time, and sonographic scanning expertise needed to perform TTE. The strong correlation between impedance and IVC size provides evidence from an external measure (TTE) that BIS tracks preload over a broad range of values (IVC sizes from ~0.5 to 4.2 cm).

ECF is an absolute quantity (liters) that depends on patient size and is therefore more informative if tracked over time for a given individual. ECF%TBW, however, is normalized (as a percentage) allowing it to be applied across populations and enabling clinically relevant stratification as show in Figure 3. ECF%TBW values >51.0%, consistent with fluid overload, were measured in the highest quartile of our HF-pop patients and in none of our CON-pop subjects. The next stratum (elevated: 46.4–51.0%) contains the HF-pop's interquartile range and the CON-pop's highest quartile; in this stratum, ECF%TBW and ECF tracking over time and reliance on symptoms/signs/labs are reasonable approaches. HF patients with ECF%TBW measurements falling into the normal stratum (41.5–46.4%) are likely compensated from a fluid status perspective because this range corresponds to the bottom three CON-pop quartiles. Lastly, ECF%TBW measurements <41.5% warrant further evaluation; for instance, repeat measurement for confirmation, and other assessments for potential volume depletion (e.g., orthostatic blood pressure measurement, blood urea nitrogen and creatinine laboratory values, etc.).

### Clinical Setting and Case Study

Given that the BIS device used in this report (Figure 1) operates while the test subject is sitting or standing without contacting metal and/or electronic objects, use in the intensive care setting is impractical. For these patients, volume status can be monitored invasively *via* pulmonary artery catheterization, and for whom fluid intake and loss is carefully tracked. BIS technology, however, may play a role in the following settings: (a) emergency departments (EDs) and urgent care centers; (b) risk stratifying HF patients at the time of hospital discharge based on the extent of residual congestion; (c) longitudinal management in clinic and skilled nursing facilities; and (d) assessing at-risk HF populations for health care managers and chief medical officers.

- Because the ED (40) and urgent care settings rely upon rapid, quantitative measures, bioimpedance-based assessment of fluid status may help facilitate triage of patients presenting with dyspnea (46). BIS measurements obtained in the ED—by serving as a point of comparison—may assist in the next phase of care if admission is required.
- HF patients, when admitted to the hospital, usually need diuresis, but knowing when sufficient decongestion has been achieved can be challenging (47). Currently, physical exams, weights, and echocardiographic measures are used to assess hydration; however, despite use of these methods, 30-day readmission rates remain high. BIS-measured ECF%TBW at the time of hospital discharge may help identify patients at high-risk of readmission owing to persistent congestion.
- In the outpatient setting, providers currently struggle with quantifying the extent of congestion. BIS may help distinguish between patients that are managed appropriately from those who may need an adjustment to their medication regimen. (47). As shown in Figure 4, BIS measurements correlate strongly with ultrasound-measured inferior vena cava size which has been used in clinic to manage diuresis and identify early fluid overload. Unfortunately, ultrasound is labor-intensive, requires a skilled operator, and is not always available.
- As more HF patients enter alternative payment models for care, objective measures of wellness are sought. A recent study of more than 500,000 patients (48) identified leg impedance measures as an independent risk factor for clinical deterioration; hence, BIS measurements at a population level may eventually help identify at-risk individuals. By directing resources to patients who pose the greatest risk for decline, healthcare systems can better meet the demand for high-yield care.

The case study presented in this report (Figure 5) is intended to provide an example of how BIS-derived fluid volume measures may be used to aid in monitoring patients with HF. It shows that ECF%TBW and ECF volume targets can be identified for heart failure patients [point (c), above]. This case also shows that patients may be discharged from hospitalizations for decompensated HF with substantial residual congestion [point (b), above]; this occurred on three occasions for this patient on study day 1 (ECF%TBW of 56.7%), day 30 (57.0%), and day 87 (54.7%).

### Limitations

As shown in Table 3, the CON-pop was younger than the HF-pop (median age 50.0 vs. 71.4 years, respectively). Aging is associated with a decrease in total body water and intracellular fluid due to decreases in muscle mass (42). Despite the difference in age, the current data-set demonstrates no significant difference between the ICF or TBW volumes measured between the two populations. This suggests that the populations are sufficiently matched for the purposes of this report. An age discrepancy would be more concerning in a randomized comparative efficacy trial; the intent of this report, however, is to use observational data to describe ECF%TBW values in health and HF. One of our goals was to identify healthy adult subjects to provide a range of normal ECF%TBW values; comorbidities increase with age, hence a younger control population is difficult to avoid. We set an age minimum of 40 years in order to age match the populations to the extent that was possible. Another objective was to describe higher ECF%TBW values characteristic of patients living with HF. In order to minimize confounders, individuals with hepatic or renal failure, nephrotic syndrome, lymphedema, and/or deep vein thrombosis/ thrombophlebitis were excluded. Consequently, ECF%TBW elevations were most likely due to fluid overload from known NYHA Class II or III HF rather than other causes. Finally, the degree of ECF%TBW elevation in our HF population is greater than what would be expected from advanced age alone. The highest ECF%TBW reported in NHANES III was 47.3 ± 2.0% for women aged 70–79 years (41), which is lower than the ECF%TBW we observed in our HF population (49.2 ± 3.2%).

Modest sample size and observational data collection are also potential limitations. Nevertheless, the number of control and heart failure ECF%TBW measurements was sufficient to yield statistically significant (*p* < 0.001) separation in distributions with non-overlapping interquartile ranges (Figure 3A). We report on 64 HF patients which, given that BIS use in HF is relatively new, is larger than HF sample sizes from previously published studies that range from five (8) to fifty (11) HF participants. The clinical strata we describe in this report represent an initial step toward quantifying fluid status in HF at the point-of-care. Refinements to account for factors such as gender, age, and HF severity/etiology should be considered as additional data are accrued. Laboratory data (e.g., hematocrit, hemoglobin, electrolytes, and natriuretic peptide levels) and detailed information regarding left ventricular ejection fraction were not collected. These limitations will be addressed by future clinical research that should also include evaluation of outcomes (e.g., 30-day readmission rates, mortality, health care costs, etc.) based on BIS-informed HF management.

## Conclusion

BIS-measured ECF%TBW values were significantly higher in HF patients as compared to adults without HF. We describe three strata of ECF%TBW that include a range of normal values (41.5–46.4%) and a threshold (>51.0%) consistent with fluid overload. Other parameters, such as ECF volume and R_0_, also differed between HF and control populations. As more data are accumulated, our results suggest that BIS measurements may provide a unique additional tool to aid in clinical decision making; however, additional BIS data controlling for confounding risk factors impacting HF will be helpful in clarifying how BIS can optimally be applied in the overall management of HF patients.

## Data Availability Statement

The datasets presented in this article are not readily available because proprietary clinical study data are reported in this article. Requests to access the datasets should be directed to bmatsubara@impedimed.com.

## Ethics Statement

The studies involving human participants were reviewed and approved by Western IRB (Central) Scripps IRB (Scripps Memorial Hospital). The patients/participants provided their written informed consent to participate in this study.

## Author Contributions

AA: conceptualization, methodology, investigation, and writing—original draft. BM: data curation, visualization, writing—original draft, and review and editing. RG: methodology, software, formal analysis, and writing—original draft. AD-B: project administration and writing—review and editing. JH: conceptualization, investigation, writing—review and editing, and supervision. All authors: contributed to the article and approved the submitted version.

## Conflict of Interest

AA, AD-B, and JH provide consultancy services to ImpediMed. BM and RG are employees of ImpediMed. As funder of this clinical research, ImpediMed provided no-cost access to bioimpedance spectroscopy devices for use in accordance with clinical trial protocols. ImpediMed also provided funding for clinical trial-related costs and third-party data management and biostatistical support.
